# LncRNA WT1-AS up-regulates p53 to inhibit the proliferation of cervical squamous carcinoma cells

**DOI:** 10.1186/s12885-019-6264-2

**Published:** 2019-11-06

**Authors:** Yunxia Zhang, Renhua Na, Xinling Wang

**Affiliations:** 1 First Department of Gynecological Radiotherapy, Affiliated Cancer Hospital of Xinjiang Medical University, Urumqi City, Xinjiang 830011 People’s Republic of China; 2 Fourth Department of Gynecology, Affiliated Cancer Hospital of Xinjiang Medical University, No. 789 Suzhou East Street, Urumqi City, Xinjiang 830011 People’s Republic of China

**Keywords:** Cervical squamous cell carcinoma, WT1-AS, p53, Prognosis, Proliferation

## Abstract

**Background:**

It has been reported that the development of cervical squamous cell carcinoma (CSCC) requires the involvement of a large number of lncRNAs. In a recent study lncRNA, WT1-AS was been characterized as a tumor-suppressive lncRNA in gastric cancer. In our study we aim to explore the involvement of WT1-AS in CSCC.

**Methods:**

Seventy-six CSCC patients (20 to 63 years, 40.1 ± 6.1 year) from the 233 CSCC patients who were admitted by the Affiliated Tumour Hospital of Xinjiang Medical University between august 2010 and January 2014. RT-qPCR, cell proliferation rate measurement, cell transfection, and western blot were carried out to analyze the samples.

**Results:**

We found that HPV infection failed to affect WT1-AS expression in CSCC tissues, while WT1-AS was down-regulated in CSCC tissues compared with non-cancer tissues. P53 was also down-regulated in CSCC tissues and positively correlated with WT1-AS. Analysis of the survival of CSCC patients revealed that low levels of WT1-AS were accompanied by poor survival. Significantly up-regulated p53 was observed after WT1-AS over-expression in CSCC cells, while p53 over-expression failed to affect WT1-AS. P53 and WT1-AS over-expression resulted in the inhibited proliferation of CSCC cells.

**Conclusion:**

Therefore, WT1-AS is down-regulated in CSCC and it may inhibit CSCC cell proliferation at least partially by up-regulating p53.

## Background

Cervical cancer is a type of human cancer characterized by its high incidence and mortality rates [1]. The popularization of human papillomavirus (HPV) vaccination and development of screening program for HPV infection result in decrease in incidence of cervical cancer during the past century [2]. However, cervical cancer is still a common type of malignancy in females [3]. It has been reported that cervical cancer cause about 300, 000 deaths every year worldwide [4]. Especially for women aged between 20 and 39 years, cervical cancer is the second leading cause of cancer-related mortalities [5]. The high mortality rate and poor treatment outcomes are mainly caused by the unknown molecular mechanism of the pathogenesis. Therefore, in-depth investigations on the molecular pathways involved in this disease are needed.

Cervical cancer is generally divided into cervical squamous cell carcinoma (CSCC) and cervical adenocarcinoma two major subtypes, and the former one accounts for about 4/5 of all cervical cancer cases [6]. Genetic alterations are the critical players in CSCC [7, 8]. Microarray analyses have revealed the dysregulation of a big number of genes during CSCC development [9]. Besides protein-coding genes, long non-coding RNAs (lncRNAs, > 200 nt) as key regulators of gene expression also participate in cancer biology by interacting with both tumor suppressive and oncogenic pathways [10, 11]. In a recent study lncRNA, WT1-AS was been characterized as a tumor-suppressive lncRNA in gastric cancer [12]. In gastric cancer, WT1-AS is down-regulated and its down-regulation promote cancer cell proliferation and invasion [12]. Our preliminary microarray showed the down-regulation of WT1-AS in CSCC and its positive correlation with p53, which is a well-studied tumor suppressor [13]. We, therefore, explored the possible interaction between WT1-AS and p53 in CSCC.

## Methods

### Research patients

We included 76 CSCC patients (all females, 20 to 63 years, 40.1 ± 6.1 year) from the 233 CSCC patients who were admitted by the Affiliated Tumour Hospital of Xinjiang Medical University between August 2010 and January 2014. Inclusion criteria: 1) the patients should be newly diagnosed CSCC patient by histopathological test, not recurrent CSCC; 2) the patients had not received any therapies for any clinical disorders within 3 months before this study. Exclusion criteria: 1) patients complicated with any other clinical disorders were excluded; 2) patients with a family history of malignancies were excluded; 3) patients with previous history of malignancies were excluded. HPV infections were detected by performing sensitive PCR. The results showed that 28 cases were HPV16 positive, 30 cases were HPV18 positive and 18 cases were negative for HPV. This study had been approved by Affiliated Tumour Hospital of Xinjiang Medical University Ethics Committee. All patients were informed with the whole operation protocol and signed informed consent.

### A 5-year follow-up study

All 76 CSCC patients were monitored for 5 years through telephone (or outpatient visit in some cases). The ones who were lost before the end of follow-up or died of other diseases, or accidence, such as traffic accidence were excluded from this study.

### Tissues

The cervical biopsy was applied to all the 76 CSCC patients before the initiation of any therapies. During biopsy, CSCC (cancer) and adjacent (within the region 5 cm around tumors) non-cancer tissues were obtained from all patients. All tissues were subjected to histopathological examinations, which were carried out by 3 experienced pathologists.

### Cells and transient transfection

To study the effects of HPV, our study includes both SiHa, an HPV positive human CSCC cell line, and C-33A, an HPV negative human CSCC cell line. Cells were obtained from ATCC (USA). We received these two cell lines in January 2019 from ATCC. These two cell lines have been authenticated by STR analysis and morphology check. According to International Cell Line Authentication Committee C-33A was mislabeled as ovarian cancer cell line, but actually it is CSCC cell line. 10/% FBS was added into Eagle’s Minimum Essential Medium to cultivate SiHa and C-33A cells under the conditions of 5% CO_2_ and 37 °C. Vectors (pcDNA3.1) expressing WT1-AS or p53 were provided by Sangon (Shanghai, China). Lipofectamine 2000 reagent (Invitrogen, USA) was used to transfect 2 μg WT1-AS or p53 expression vectors or pcDNA3.1 empty vectors (negative control, NC) into 10^6^ SiHa or C-33A cells. In this experiment, cells without any transfections were used as control (C).

### RNA extractions and RT-qPCR

RNAzol RT (Sigma-Aldrich, USA) was mixed with tissues (ground in liquid nitrogen before use) and cultivated SiHa and C-33A cells to extract total RNAs. After DNase I digestion, QuantiTect Reverse Transcription Kit (QIAGEN China, Shanghai, China) was used to carry out reverse transcriptions to synthesize cDNA. All qPCR mixtures were prepared using QuantiTect SYBR Green PCR Kit QIAGEN China, Shanghai, China) to detect the expression of WT1-AS and p53. Endogenous controls for WT1-AS and p53 were 18S rRNA and GAPDH, respectively. All reactions were replicated 3 times and Ct values were processed using the 2^-ΔΔCT^ method.

### Cell proliferation rate measurement

SiHa and C-33A were collected at 24 h after transfections, and 3 × 10^4^ cells were mixed with 1 ml Eagle’s Minimum Essential Medium (% 10 FBS) to prepare single-cell suspensions. To test cell proliferation ability, all cells were transferred to a 96-well plate, 100 μl per well. Under the conditions of 5% CO_2_ and 37 °C, cells were cultivated and addition of 10 μl CCK-8 solution (Sigma,-Aldrich, USA) was performed every 24 h for 4 times. To reflect cell proliferation, OD values (450 nm) were measured.

### Western-blot

SiHa and C-33A were collected at 24 h after transfections, and 10^6^ cells were mixed with 1 ml RIPA solution (Thermo Fisher Scientific) to extract total proteins. Protein samples with high quality were subjected to 10% SDS-PAGE gel electrophoresis after denaturing. After that, proteins were transferred to PVDF membranes through the semi-dry method, and 5% milk (non-fat) was used to incubate the membranes for 2 h at 22 °C. After that GAPDH (ab37168, 1:1500, Abcam) and p53 (ab131442, 1:1500, Abcam) primary rabbit polyclonal antibodies were used to incubate with the membranes overnight at 4 °C. After that, goat anti-rabbit IgG-HRP secondary antibody (MBS435036, 1:1500, MyBioSource) was used to further incubate with membranes at 22 °C for 2 h. Signals were developed using ECL (Thermo Fisher Scientific) and Image J v1.46 software was used to develop signals.

### Statistical analysis

Experiments were repeated 3 times to calculate mean values. Correlations were analyzed by linear regression. The 76 CSCC patients were grouped into low (*n* = 40) and high (*n* = 36) WT1-AS level (in CSCC tissues) groups according to Youden’s index (cutoff value = 2.09). Based on survival data of two groups, survival curves were plotted using the Kaplan-Meier method and compared using log-rank test. The paired t-test was used to analyze differences between non-cancer tissues and CSCC tissues. One-way ANOVA and Tukey test were used to analyze differences among different groups of patients and different cell transfection groups. *p* < 0.05 indicated statistical significance.

## Results

### WT1-AS was affected by CSCC but not by HPV infections

**Fig. 1:**
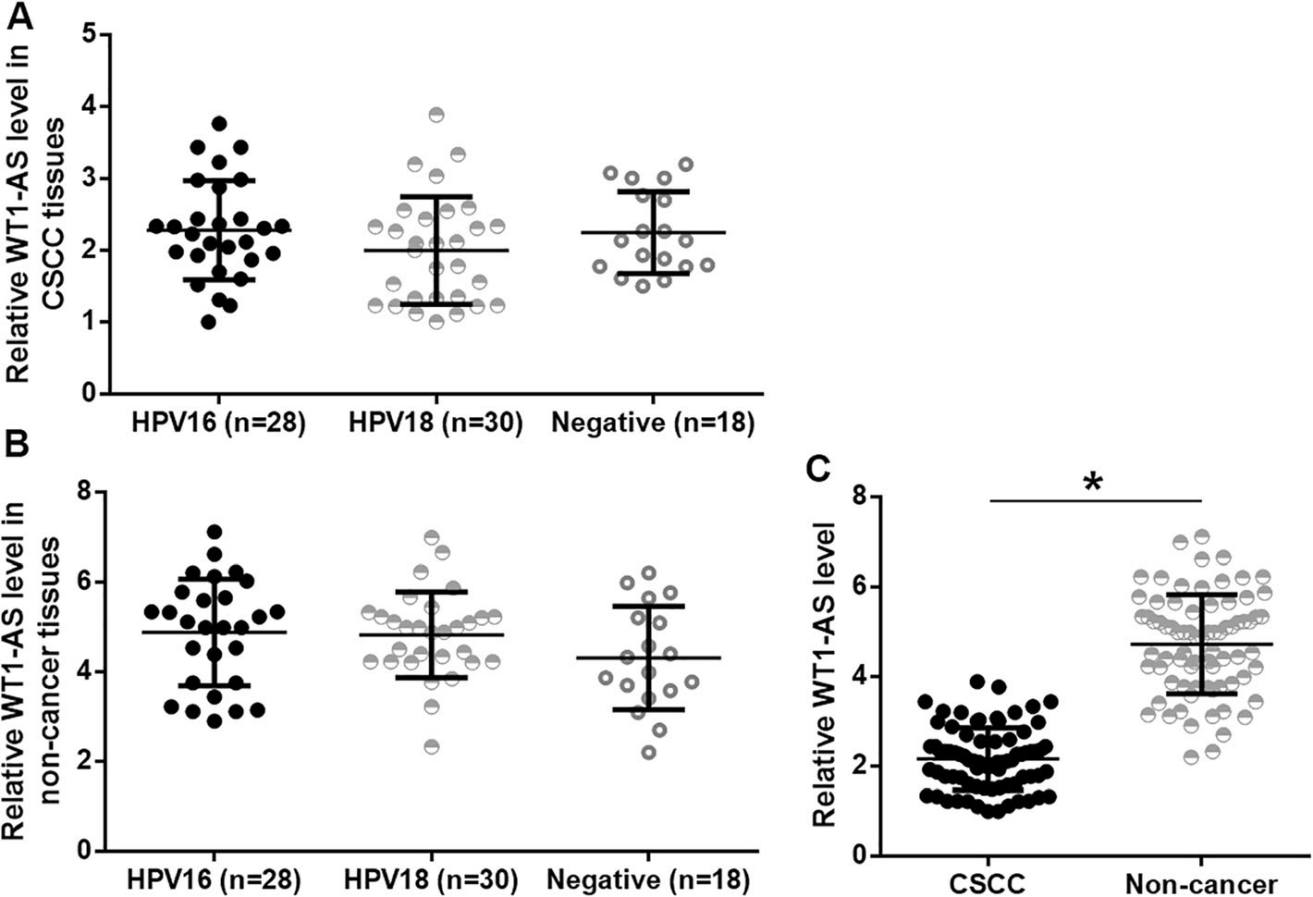
WT1-AS was affected by CSCC but not by HPV infections. WT1-AS expression was analyzed by qPCR. Expression data analyzed by one-way ANOVA and Tukey test showed that expression levels of WT1-AS in CSCC tissues (**a**) and non-cancer tissues (**b**) were not significantly different among 3 groups of patients. Expression data analyzed by paired t-test showed that levels of WT1-AS were significantly lower in CSCC tissues than in non-cancer tissues (**c**). All PCR reactions were repeated three times and mean values were presented. (*, *p* < 0.05)

WT1-AS in CSCC and non-cancer tissues of CSCC patients (*n* = 76) was detected by performing RT-qPCR. Among the 76 patients, 28 cases were HPV16 positive, 30 cases were HPV 18 positive and 18 cases were negative for HPV. Expression levels of WT1-AS were compared among these 3 groups by performing one-way ANOVA and Tukey test. It was observed that expression levels of WT1-AS in CSCC tissues (Fig. 1a) and non-cancer tissues (Fig. 1b) were not significantly different among 3 groups of patients. Expression levels of WT1-AS were compared between CSCC and non-cancer tissues by performing the paired t-test. It was observed that levels of WT1-AS were significantly lower in CSCC tissues than in non-cancer tissues (Fig. 1c, *p* < 0.05).

### Low levels of WT1-AS were accompanied by poor survival

**Fig. 2:**
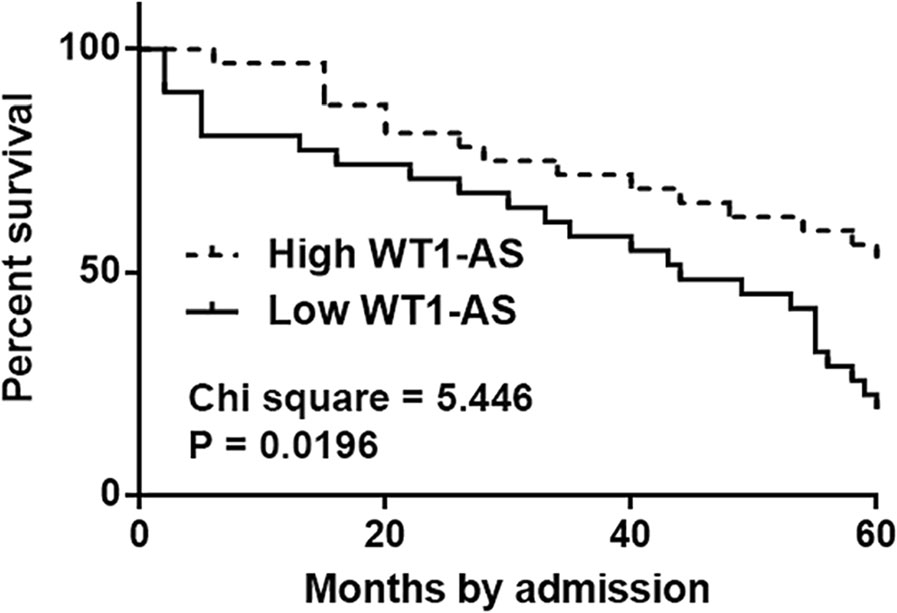
Low levels of WT1-AS were accompanied by poor survival. The 76 CSCC patients were grouped into low (*n* = 40) and high (*n* = 36) WT1-AS level (in CSCC tissues) groups. Based on survival data of two groups, survival curves were plotted using Kaplan-Meier method and compared using log-rank test

The 76 CSCC patients were grouped into low (*n* = 40) and high (*n* = 36) WT1-AS level (in CSCC tissues) groups. Based on survival data of two groups, survival curves were plotted using the Kaplan-Meier method and compared using log-rank test. It was observed that the overall survival rate of low WT1-AS level group was significantly lower than that of high WT1-AS level group (Fig. 2).

### WT1-AS was positively correlated with p53 in CSCC tissues

**Fig. 3:**
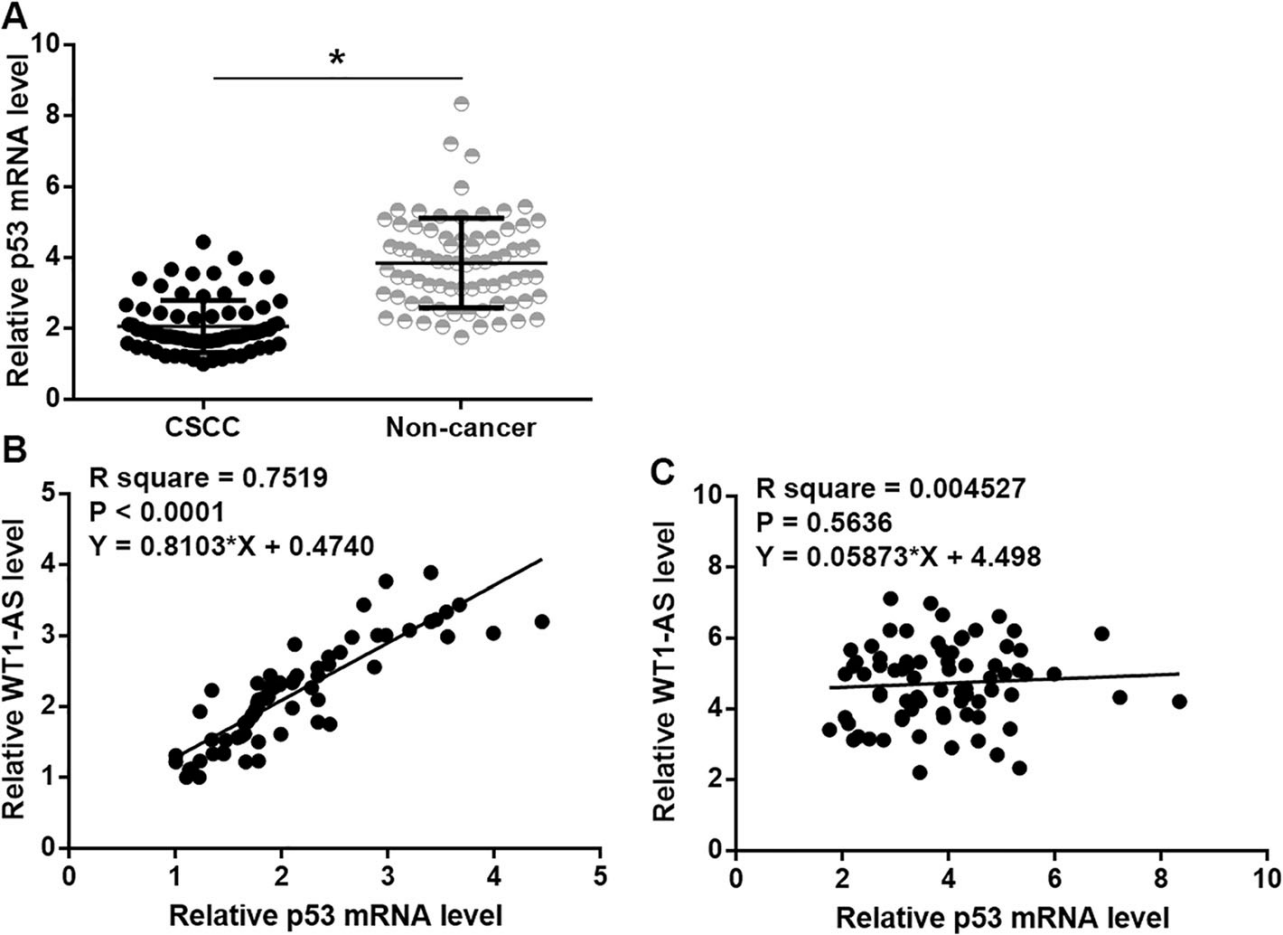
WT1-AS was positively correlated with p53 in CSCC tissues. Analysis of p53 expression data by performing paired t-test analysis showed that levels of p53 mRNA were significantly lower in CSCC tissues than in non-cancer tissues of the 76 CSCC patients (**a**). PCR reactions were repeated 2 times and mean values were presented.(*, *p* < 0.05). Linear regression analysis showed that p53 mRNA and WT1-AS were significantly and positively correlated with each other in CSCC tissues (**b**), but not in non-cancer tissues (**c**)

RT-qPCR was also performed to investigate the expression pattern of p53. Paired t-test analysis showed that levels of p53 mRNA were significantly lower in CSCC tissues than in non-cancer tissues of 76 CSCC patients (Fig. 3a, *p* < 0.05). Correlations between p53 mRNA and WT1-AS were analyzed by performing linear regression. In CSCC tissues, p53 mRNA and WT1-AS were significantly and positively correlated with each other (Fig. 3b). In non-cancer tissues, the correlation between these two factors was not significant (Fig. 3c).

### WT1-AS up-regulated p53 and inhibited CSCC cell proliferation

**Fig. 4:**
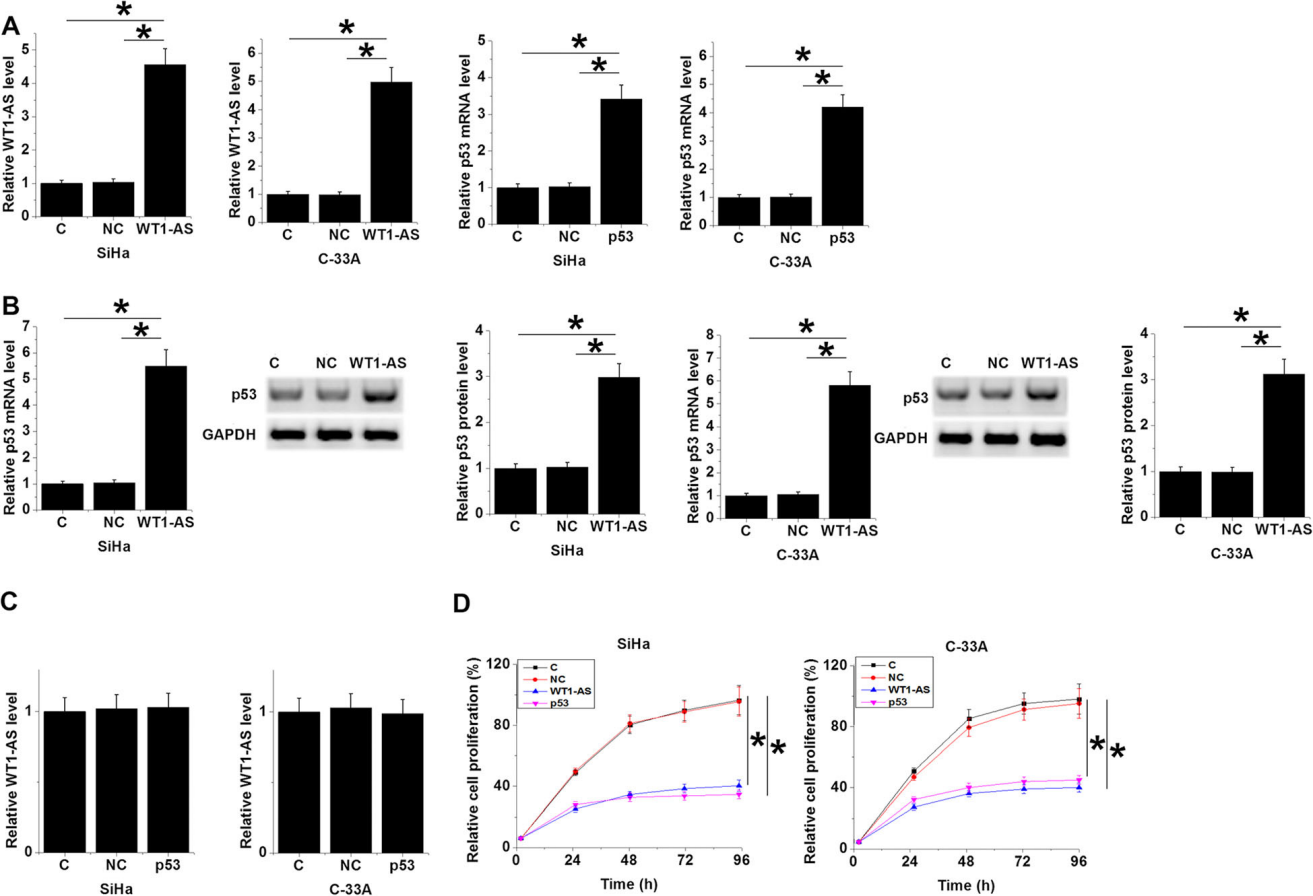
WT1-AS up-regulated p53 and inhibited CSCC cell proliferation. SiHa and C-33A cells were transfected with WT1-AS and p53 expression vectors, followed by the confirmation of over-expression of WT1-AS and p53 by qPCR at 24 h post-transfection (**a**). In addition, qPCR and western blot analysis showed significantly up-regulated p53 after WT1-AS over-expression at both mRNA and protein levels in both SiHa (left) and C-33A (right) cell line (**b**). In section (**b**), for each cell line left panel showed the normalized expression level of p53 mRNA, middle panel showed the representative western blot images, and right panel showed the normalized western blot data. In contrast, qPCR analysis showed that p53 over-expression failed to affect WT1-AS (**c**). Moreover, p53 and WT1-AS over-expression resulted in inhibited proliferation of CSCC cells (**d**). All experiments were repeated 3 times and mean values were presented. (*, *p* < 0.05)

WT1-AS and p53 expression vectors were transfected into SiHa and C-33A cells. It was observed that expression levels of WT1-AS and p53 were significantly increased at 24 h after transfections comparing to NC and C groups (Fig. 4a, *p* < 0.05). In addition, significantly up-regulated p53 was observed after WT1-AS over-expression at both mRNA and protein levels (Fig. 4b, p < 0.05), while p53 over-expression failed to affect WT1-AS (Fig. 4c). Moreover, p53 and WT1-AS over-expression resulted in the inhibited proliferation of CSCC cells (Fig. 4d, *p* < 0.05).

## Discussion

The roles of WT1-AS have only been characterized in gastric cancer [12]. This study investigated the function of WT1-AS in CSCC and explored its clinical significance. We also observed that WT1-AS may inhibit CSCC by up-regulating tumor-suppressive p53.

Accurate prognosis is always critical for the survival of many types of cancers due to the low early diagnostic rate and difficulties in developing sensitive and specific early diagnostic markers [14]. In recent years, circulating biomarkers, such as lncRNAs or miRNAs in blood have been widely used in disease prediction [15]. However, our study failed to detect WT1-AS blood in most CSCC patients. This is possibly due to its low level of blood. Our future study will develop sensitive PCR program to detect WT1-AS in blood. The follow-up data suggested that low levels of WT1-AS in CSCC are accompanied by poor survival of CSCC patients, suggestive of its prognostic values.

HPV infections, such as HPV16 and 18 infections are the major causes of CSCC [16]. In the present study, we observed no significant effects of HPV infections on WT1-AS expression in both CSCC tissues and non-cancer tissues. We speculate that WT1-AS may participate in CSCC through HPV- independent pathways.

WT1-AS was down-regulated in CSCC, indicating its involvement in this disease. Our over-expression experiments showed suppressed proliferation of both HPV-positive and -negative cells after WT1-AS over-expression. Those data suggested the tumor-suppressive roles of WT1-AS in CSCC. p53 is a well-studied tumor suppressor [13]. p53 inhibits cancer development through the regulation of cancer cell behaviors, such as proliferation in many types of cancer including CSCC [17]. We also observed suppressed CSCC cell proliferation after p53 over-expression. It is known that p53 may interact with lncRNAs to play its roles in cancer biology [18]. We in this study proved that WT1-AS was an upstream activator of p53. But the interaction between WT1-AS and p53 may be indirect due to the lack of significant correlation between them in non-cancer tissues.

## Conclusion

In conclusion, WT1-AS is down-regulated in CSCC and it may inhibit CSCC cell proliferation at least partially by up-regulating p53.

## Data Availability

The datasets used and/or analyzed during the current study are available from the corresponding author on reasonable request.

## Abbreviations

CSCC: Cervical squamous cell carcinoma
HPV: Human papillomavirus
lncRNAs: long non-coding RNAs

## References

[1] Ferlay J, Soerjomataram I, Dikshit R, Eser S, Mathers C, Rebelo M (2015). Cancer incidence and mortality worldwide: sources, methods and major patterns in GLOBOCAN 2012. Int J Cancer.

[2] Hildesheim A, Gonzalez P, Kreimer AR, Wacholder S, Schussler J, Rodriguez AC (2016). Impact of human papillomavirus (HPV) 16 and 18 vaccination on prevalent infections and rates of cervical lesions after excisional treatment. Am J Obstet Gynecol.

[3] Siegel RL, Miller KD, Jemal A (2016). Cancer statistics, 2016. CA Cancer J Clin.

[4] Torre LA, Bray F, Siegel RL, Ferlay J, Lortet-Tieulent J, Jemal A (2015). Global cancer statistics, 2012. CA Cancer J Clin.

[5] Siegel RL, Miller KD, Jemal A (2018). Cancer statistics, 2018. CA Cancer J Clin.

[6] Berman NR, Koeniger-Donohue R. Cervical cancer. In: Advanced Health Assessment of Women: Clinical Skills and Procedures. New York: Springer Publishing Co Inc; 2018. p. 431.

[7] Cancer Genome Atlas Research N, Albert Einstein College of M, Analytical Biological S, Barretos Cancer H, Baylor College of M, Beckman Research Institute of City of H (2017). Integrated genomic and molecular characterization of cervical cancer. Nature.

[8] Umayahara K, Numa F, Suehiro Y, Sakata A, Nawata S, Ogata H (2002). Comparative genomic hybridization detects genetic alterations during early stages of cervical cancer progression. Genes Chromosomes Cancer.

[9] Narayan G, Bourdon V, Chaganti S, Arias-Pulido H, Nandula SV, Rao PH (2007). Gene dosage alterations revealed by cDNA microarray analysis in cervical cancer: identification of candidate amplified and overexpressed genes. Genes Chromosomes Cancer.

[10] Yang G, Lu X, Yuan L (2014). LncRNA: a link between RNA and cancer. Biochim Biophys Acta.

[11] Schmitt AM, Chang HY (2016). Long noncoding RNAs in Cancer pathways. Cancer Cell.

[12] Du T, Zhang B, Zhang S, Jiang X, Zheng P, Li J (2016). Decreased expression of long non-coding RNA WT1-AS promotes cell proliferation and invasion in gastric cancer. Biochim Biophys Acta.

[13] Muller PA, Vousden KH (2013). p53 mutations in cancer. Nat Cell Biol.

[14] Kourou K, Exarchos TP, Exarchos KP, Karamouzis MV, Fotiadis DI (2015). Machine learning applications in cancer prognosis and prediction. Comput Struct Biotechnol J.

[15] Hyun KA, Kim J, Gwak H, Jung HI (2016). Isolation and enrichment of circulating biomarkers for cancer screening, detection, and diagnostics. Analyst.

[16] de Martel C, Plummer M, Vignat J, Franceschi S (2017). Worldwide burden of cancer attributable to HPV by site, country and HPV type. Int J Cancer.

[17] Li Q, Li X, Wang C (2016). Uc.206 regulates cell proliferation and apoptosis by targeting P53 in cervical cancer cells. Neoplasma.

[18] Mao C, Wang X, Liu Y, Wang M, Yan B, Jiang Y (2018). A G3BP1-interacting lncRNA promotes Ferroptosis and apoptosis in Cancer via nuclear sequestration of p53. Cancer Res.

